# Veridical mapping in savant abilities, absolute pitch, and synesthesia: an autism case study

**DOI:** 10.3389/fpsyg.2014.00106

**Published:** 2014-02-18

**Authors:** Lucie Bouvet, Sophie Donnadieu, Sylviane Valdois, Chantal Caron, Michelle Dawson, Laurent Mottron

**Affiliations:** ^1^Laboratoire de Psychologie et Neurocognition, CNRS UMR 5105Grenoble, France; ^2^Université Lille 3 - Charles de GaulleLille, France; ^3^Université de SavoieChambéry, France; ^4^Centre National de la Recherche ScientifiqueParis, France; ^5^Centre d’Excellence en Troubles Envahissants du Développement de l’Université de Montréal, Hôpital Rivière-des-PrairiesMontréal, Canada

**Keywords:** autism, synesthesia, savant abilities, cognition, veridical mapping

## Abstract

An enhanced role and autonomy of perception are prominent in autism. Furthermore, savant abilities, absolute pitch, and synesthesia are all more commonly found in autistic individuals than in the typical population. The mechanism of veridical mapping has been proposed to account for how enhanced perception in autism leads to the high prevalence of these three phenomena and their structural similarity. Veridical mapping entails functional rededication of perceptual brain regions to higher order cognitive operations, allowing the enhanced detection and memorization of isomorphisms between perceptual and non-perceptual structures across multiple scales. In this paper, we present FC, an autistic individual who possesses several savant abilities in addition to both absolute pitch and synesthesia-like associations. The co-occurrence in FC of abilities, some of them rare, which share the same structure, as well as FC’s own accounts of their development, together suggest the importance of veridical mapping in the atypical range and nature of abilities displayed by autistic people.

## INTRODUCTION

Savant abilities involve a marked contrast within the same individual, in whom apparent intellectual or developmental disabilities co-exist with strong, sometimes outstanding, specific talents. The prevalence of savant or exceptional abilities in autism is understudied and likely has been underestimated, with popularly reported figures such as 1 in 200 (Hermelin, 2001) falling well short of current findings [approximately 1 in 3; (Howlin et al., 2009)]. To date the savant literature has concentrated on abilities in a few general areas thought to be characteristic, for example drawing (Mottron and Belleville, 1993; Wallace et al., 2009), musical skills (Sloboda et al., 1985; Young and Nettelbeck, 1995; Heaton et al., 2008), and calendrical calculation or other types of memory (Mottron et al., 2006b; Thioux et al., 2006; Neumann et al., 2010), as well as hyperlexia (Nation, 1999; Newman et al., 2007). However, it should be noted that many other savant abilities or entire areas of savant ability may exist but are yet to be adequately studied (e.g., estimation; Soulières et al., 2010). Given the high occurrence of savant ability in autism, and reciprocally the high prevalence of autism or autistic traits among savants (Heaton and Wallace, 2004), the emergence of savant abilities is linked with some specificity to the autistic mind and brain.

Using the weak central coherence theory framework (Happé and Frith, 2006), Happé and Vital (2009) proposed that an autistic processing bias toward local information may predispose these people to talent. Based on parental report only, they observed that items considered related to a detail-focused cognitive style were more pronounced among children with “special abilities” than among those without. However, this cannot explain the acquisition of savant abilities in autism, and the concept of local bias itself is ill-defined and intrinsically multi-level. Baron-Cohen et al. (2009) proposed that local bias or detail-focus associated with another concept, hyper-systemizing, might account for the predisposition of autistic^1^ people to talent. Systemizing is defined as “the drive to analyze or construct systems”; systems in turn are sets of information following rigid, predictable “if *p*, then *q*” rules. For instance, systemizing can account for superior performances in some folk physics tests (Baron-Cohen et al., 2001; Binnie and Williams, 2003). However, while rules may govern common areas of savant abilities, such as 3-D drawing, music, calculation, and the decoding of written language, these areas may also be characterized by irregularities and unpredictability, as in English orthography or musical improvisation.

The enhanced perceptual functioning (EPF) model (Mottron et al., 2006a) provides another trend of thought, in which an enhanced role and autonomy of perception may be prominent in what is specific to the autistic mind, and particularly, in the acquisition of savant abilities. Existing evidence for superior perceptual processes in autism comes primarily from results of visual and auditory tasks (for reviews, see Simmons et al., 2009; O’Connor, 2012), including those which are not performed perceptually by typical participants (i.e., working memory task, Koshino et al., 2005) and/or are considered “high-level” (i.e., matrix reasoning Soulières et al., 2009). Enhanced perception and manipulation of specific materials might predispose autistic individuals to develop savant abilities, given the opportunity, as in 3-D drawing or musical improvisation. However, as with proposals based on detailed-focused cognitive styles and/or hyper-systemizing, there is insufficient evidence as to how enhanced perception leads to the development of savant abilities (Dawson et al., 2008).

To address this shortcoming, the EPF model has recently been extended via a proposed veridical mapping (VM) mechanism, which represents an attempt to address how and why savant and other related unusual abilities develop in autism (Mottron et al., 2009; Mottron et al., 2013). VM is a capacity to detect regularities within and across isomorphic structures (i.e., structures sharing perceptual or structural similarity), at multiple scales. The materials involved in savant abilities are often structured human codes (e.g., written, arithmetical, and musical structures) which are also multi-level and redundant (i.e., sentences composed of words composed of letters, songs composed of melodies composed of notes, years composed of months composed of days). These materials exist across multiple scales, from very low-level or simple to very high-level or complex, and can be seen as highly isomorphic.

Veridical mapping in autism may arise from the combined effect of enhanced low-level perceptual abilities and superior mid-level ability in manipulating complex patterns. As a mechanism, VM would allow autistics to flexibly detect complex repetitive occurrences within human codes, while operating a parallel mapping of their constituents with other structures sharing some perceptual or structural similarity. It allows the memorization of the coupling between homologous elements of these structures, as in grapheme-phoneme coupling evident in hyperlexia. A basic example of VM in autism can be found in the autobiographical account of GT, a 9-year-old autistic boy, who could make outstanding weight, height, and distance estimations. GT reported that he used the recurrent mapping of a weight of a cereal bar of 35 g to estimate weights under 10 kg, which was confirmed by his contrasting accuracy in estimation of weights superior versus inferior to this amount (Soulières et al., 2010).

We have also proposed (Mottron et al., 2013) that the same VM mechanism might also account for the plausibly superior prevalence of synesthesia (Baron-Cohen et al., 2013; Neufeld et al., 2013) and absolute pitch (DePape et al., 2012) in autism. This proposal draws on multiple observed similarities or overlaps connecting the two abilities. Synesthesia is condition where “attribute of a stimulus (e.g., its sound, shape, or meaning) may inevitably lead to the conscious experience of an additional attribute” (Ward, 2013, p.50). This definition has been quite useful to describe the most studied forms of synesthesia, such as colored perception of alphabet letters or musical notes. Recently this definition has been questioned due to the rising number of different forms of synesthesia (61 according to Cytowic et al., 2009), some of which involve more elaborate cognitive traits. For example, some people associate a specific personality with a certain number or letter (Simner and Holenstein, 2007; Smilek et al., 2007), which is called ordinal linguistic personification (OLP). Thus, Simner (2012) recently proposed a new definition: “Synaesthesia is characterized by the pairing of a particular triggering stimulus with a particular resultant experience.” (p. 12).

Absolute pitch is defined as the ability to name or otherwise indicate notes without reference to an external standard. Synesthesia and absolute pitch share some common neural mechanism (Loui et al., 2012). The association between note and label in absolute pitch possessors is automatic (Akiva-Kabiri and Henik, 2012; Schulze et al., 2012), as is the case with associations in synesthesia (Ward, 2013). Genetic linkage and co segregation between absolute pitch and synesthesia has also been observed (Gregersen et al., 2013). Synesthesia possessors display characteristics neighboring those observed in autism, such as superior perceptual capacities (Banissy et al., 2009; Goller et al., 2009) and superior mental rotation in time-space synesthetes (Simner et al., 2009; Brang et al., 2010, 2013) who also show memory benefits that could be linked to savant abilities (Simner et al., 2009). Further, the cross modal retrieval of a concurrent element (i.e., the resultant synesthetic experience) when its inducer (i.e., the element that elicits synesthesia) is perceived in synesthesia has similarities with the possible role in savant abilities of redintegration, which is the non-strategic recall of a missing element in the presence of its homolog (Mottron et al., 2013). First-hand accounts document the co-occurrence of synesthesia and savant syndrome, as in the case of Daniel Tammet (Bor et al., 2007). It has been suggested by some authors that when autism and synesthesia co-occur, the probability of savant syndrome is increased (Baron-Cohen et al., 2007; Simner et al., 2009). These authors proposed that an over-rehearsal related to the repetitive nature of interest in the autistic population can lead to the development of savant abilities.

In this paper we present a case study of FC, a savant autistic adult, which may cast a new light on the nature of this link. FC is exceptional at several levels. First, he possesses multiple perceptual and non-perceptual savant abilities, among which some (e.g., calendar calculation) are in classical savant areas but others (e.g., absolute pitch) are marginally if at all considered to be savant abilities. Second, despite limited verbal abilities, FC is able to provide spoken accounts of some of his methods, providing exceptional information on the way they progress with time. Third, FC also possesses some synesthesia-like associations. We will report a description and empirical study of FC’s abilities as well as his own accounts of the acquisition of these abilities.

## CASE REPORT

### DEVELOPMENTAL HISTORY

FC was born by cesarean section to a 23-year-old mother following an uncomplicated 42-week pregnancy. He weighed 2.62 kg and measured 47 cm at birth. He has one older brother and one younger sister who are both typically developing. His mother is the youngest of a family of three and his father is the 9th child from a family of 10. One cousin on his father’s side presents an intellectual disability and another suffers from major thalassemia; there was nothing relevant to report on his mother’s side. Both parents operate a restaurant, although his father is trained as an electrician.

FC’s first year of life was marked by repeated ear infections with bilateral myringotomy and, retrospectively, atypical calmness and hypo-activity. Motor milestones were unremarkable. At the age of two, his parents suspected atypical development based on absence of speech, as well as presence of swaying and excessive quietness. A 2-week stay in a neurological hospital revealed normal physical and auditory functions, but a suspected childhood psychosis, a frequent diagnosis for French autistic toddlers at the time. FC started attending a nursery school at the age of three. By 5 years of age, FC still did not utter a single word but used his parents’ hands to communicate his needs, or emitted small noises. His first word was his brother’s name and by age six, his vocabulary was only 10 words. At this age, immediate echolalia appeared and FC’s spoken language then evolved from echolalia to standard use of speech. Between 5 and 10 years, he received regular speech therapy support, attended a mainstream school, and followed a specific program in an outpatient clinic for children with learning disability. FC was in this period able to read without apparent understanding of what he was reading, but by the age of 10 was able to read, write, and count typically. He used to cover his ears with his hands or run away when he was confronted with unknown or loud sounds.

The diagnosis of autism was given at 11 years and 7 months by a professional clinician, on the basis of above-threshold past and current ADI (Le Couteur et al., 1989) algorithm scores in the four areas of social interaction, communication, restricted interests and repetitive behaviors and age of concern. His CARS score (Schopler et al., 1980) at this age was 31, above the threshold for autism. As a teen, he was integrated in a specialized school for neurodevelopmental disorders.

FC was 21 years old at the beginning of testing and 26 years old at the time of writing. He is now working in an establishment for disabled people, doing repetitive work. He gives brief eye contact and socially oriented smiles, but in an atypical way, and also experiences tics (hissing like a snake or looking at his watch). When he speaks, under a certain insistence, he uses stereotyped verbal expressions with some verbal apraxia. An intellectual assessment using WAIS-III at the age of 22.2 years (see **Table 1**) indicated verbal comprehension and processing speed index scores in the intellectual disability range (under the 2nd percentile), but normal-range perceptual organization and working memory index scores. In contrast, FC obtained a raw score of 55 (95th percentile, 1985 norms), in the range of superior intelligence, on Raven’s Progressive Matrices, a major test of fluid intelligence which may provide the best estimate of autistic intelligence.

**Table 1:** FC’s IQ profile from the WAIS-III.

Tests	Score
**Verbal comprehension index**	**60**
Similarities	3
Vocabulary	2
Information	1
Comprehension	4
**Working memory index**	**75**
Arithmetic	7
Digit span	10
Letter number sequencing	1
**Perceptual Organization Index**	**93**
Picture completion	6
Block design	10
Picture arrangement	2
**Processing speed index**	**54**
Matrix reasoning	11
Digit symbol-coding	2
Symbol search	2
**Verbal IQ**	**66**
**Performance IQ**	**76**
**Full Scale IQ**	**68**

During interviews, FC was able to answer questions about how he acquired his unusual abilities and the mechanisms that underlie them. We video-recorded these interviews and transcribe here the explanations he gave us, along with ecological descriptions of behaviors related to his abilities, and test results in four areas: absolute pitch, memory skills, computational ability, and synesthesia. Because FC possesses tics and has difficulty in understanding rapid instructions, tests were developed to evaluate the stability of the described associations, using discrimination tasks. Therefore, while we provide his reaction times (RTs) on different tests, as they may to some extent reflect how he processes information, they cannot be taken as unambiguous or definitive in this respect.

### ABSOLUTE PITCH

#### Related behaviors

FC possesses absolute pitch, that is, he is able to name a note without external reference. Despite never playing the domestic organ before the age of nine, FC’s parents noticed that at this age he was able to reproduce a song on the organ immediately after hearing it on the radio. After this age, FC displayed numerous music- and sound-related occupations and activities. For example, he possessed a small electronic piano that he brought everywhere. He also recorded his voice and what he was playing on a portable recorder and played it repetitively. He tried different instruments, including the guitar, flute, accordion, violin, and drums, but the piano was his favorite instrument. FC plays both classical and modern songs, such as Bach’s Toccata and Fugue in D minor BWV 565 and the John Lennon song “Imagine.” He also composed a melody, mostly of consonant third intervals, which he named “Repezik” and played over and over – “because that made me very pleased,” he reported. FC took a few piano lessons at the age of 10, but at the time was overtly more interested in the sound of the piano than in playing the instrument. FC is now taking piano lessons and is attracted by composers like Bach or Handel. According his teacher, he possesses superior abilities to an adult amateur player, particularly his memory for and immediate reproduction of melodies. He plays with his two hands independently but has more difficulty with rhythm.

#### Self-report

**FIGURE 1:**
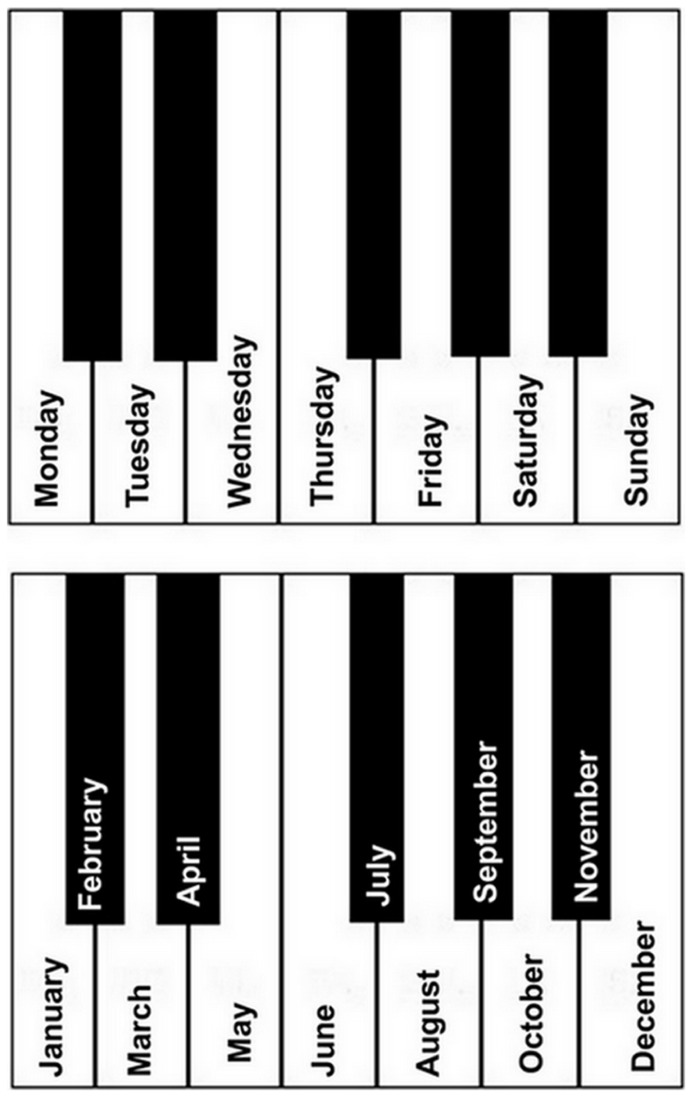
**Representation of FC’s associations of weekdays (top) and months (bottom) with musical notes**.

We asked FC how he knew that the note C corresponds to the C key. FC showed us the keyboard. Starting with the C key, he explained “C is Monday, D is Tuesday, E is Wednesday, F is Thursday, G is Friday, A is Saturday, and B is Sunday.” Then he added, while pointing to the right proximal C key on the keyboard “Next C is next Monday” and pointing to the next C key on the keyboard, said “next C and next Monday”. Then he pointed to the left proximal C key and explained “last Monday,” and the next left C key, indicating “Monday before.” We then asked him to which days the black keys correspond. Starting with the C key, and while pointing to each of the 12 keys composing an octave, he said “January, February, March, April, May, June, July, August, September, October, November, December. And everything from there (the C key) until there (the next right B flat key), is a year. And from there (C) until there (the next right B flat key), these are two years. And from there (C key three octaves higher) until there (next B flat key), it makes five years.” When we asked him whether somebody taught him or he developed this by himself, he replied, “Me, because it’s in my head.” An illustration of this association is provided in **Figure 1**.

#### Test results

FC’s receptive absolute pitch was assessed through the identification of 60 musical notes without reference note (Vangenot, 2000). Each note lasted 1000 ms, with an ISI of 2000 ms. In order to prevent the use of relative pitch, there was more than one octave between each note. He orally gave the name of the note. Absolute pitch was also tested in production, by asking him to sing 12 notes when provided with their names. His production was recorded and compared to reference musical notes. Both FC’s identification and production were 100% correct. His capacity to identify notes embedded in chords was also tested. There were three trials per chord of different numbers of notes, starting by three and up to six notes (12 different trials). His identification of notes within chords up to four notes was 100% correct. For chords composed of more than 4 notes, FC did not make any false identifications but reported only a subsample of the notes composing the chord.

FC’s idiosyncratic note-calendar mapping was tested receptively using 14 1000 ms notes ranging from B3 flat to D5 sharp, produced with Final® software, and a computer screen presentation of days of the week and months of the year. In each trial, a day (or a month) was visually presented, together with a note presented aurally. The association could be either congruent or incongruent per FC’s system of mapping. Days and months were visually presented (in 24-point Arial font) in the center of the screen and remained presented until a response was given. Trials were interleaved with a blank screen for 2000 ms.

There were 168 trials in the notes-days experiment. According to FC’s mapping, half (84) were congruent and half were incongruent. In the incongruent trials, days were presented with a note that could be higher or lower by one tone compared to the congruent note. For example, Thursday, associated with the F note in FC’s mapping, was associated with the E note and the G note in incongruent trials. Similarly, there were 192 trials in the notes-months experiment, half (96) congruent and half incongruent according to FC’s mapping. In the incongruent trials, months were presented with a note either one semitone higher or lower than the congruent note. For example, March was associated with the D note in FC’s mapping and therefore in congruent trials, while March was presented with a C sharp or D sharp note in incongruent trials. Days and months were pseudo-randomly selected in the material list to avoid the consecutive presentation of the same day or month. FC was asked if the sound presented corresponded to the correct day or month and responded by pressing with his right hand a green button for accurate (congruent) correspondences and a red button for the inaccurate (incongruent) correspondences. The experiment was run with PsychoPy software (Peirce, 2007).

FC was able to correctly identify 100% of the congruent and incongruent day-note associations (see **Table 2**). His RT for congruent trials (after removing 3SD outliers, which represented 4.76% of the trials) was 2.369 s (SD = 0.446). His mean RT for incongruent trials (after removing 3SD outliers which represented 1.19 % of the trials) was 2.309 s (SD = 0.475). There was no difference between congruent and incongruent conditions on mean RT (*p* > 0.4). In the month-note experiment, FC made 6 mistakes and was able to identify correctly 96.8% of trials in the congruent condition, and 96.8% of trials in the incongruent condition. His mean RT for congruent trials (after removing errors and 3SD outliers, which represented 14.58% of the trials) was 2.397 s (SD = 0.428). His mean RT for incongruent trials (after removing errors and three SD outliers, which represented 9.37% of the trials) was 2.369 s (SD = 0.418). There was no difference between congruent and incongruent conditions on mean RT (*p* > 0.5). FC can therefore redintegrate the missing element in pairs of homolog elements with a speed and accuracy poorly compatible with a strategic or algorithmic computation.

**Table 2:** Summary of FC’s performance.

	Score	Reaction time in s (SD)
**Absolute pitch**
Pitch identification	60/60	
Pitch production	12/12	
Chord disembedding	6/12	
*Day – note mapping*
Congruent	84/84	2.369 (0.446)
Incongruent	84/84	2.309 (0.475)
*Month – note mapping*
Congruent	89/92	2.397 (0.428)
Incongruent	89/92	2.369 (0.418)
**Calendar calculation**
Future date	9/10	
Past date	9/10	
Reverse question	16/20	
**Computation ability**
*Number – time mapping*
Congruent	40/40	6.244 (2.14)
Incongruent	40/40	6.725 (2.01)
Synesthesia-like manifestations
*Number – valence mapping*
Congruent	96/96	2.266 (0.752)
Incongruent	92/96	2.033 (0.471)

### CALENDAR CALCULATION

#### Related behaviors

FC is a calendar calculator: he can tell which day of the week corresponds to a date (day, month, year). His parents were unaware of this ability before our study, but they reported that FC had been interested in calendars, dates, and time since the age of six.

#### Self report

We asked FC how he performed calendar calculation. “I’m doing like this,” he said and then he turned toward a piano, pointed to the keys, and said “I’m thinking with musical notes. C is Monday, D is Tuesday, E is Wednesday, F is Thursday, G is Friday, A is Saturday, and B is Sunday. And these seven notes are one week.” We asked him if he thinks using the sound of the note or the name of the note. He said “I think it’s like musical note, with the name of the musical note. Like this it’s easier.” It seems therefore that he uses a synesthetic correspondence as a support for his calendrical computation ability.

#### Test results

In 2011, we questioned FC on the weekday of 10 past (from year 1993 to year 2011) and 10 future (from year 2013 to year 2031) dates. Questions did not involve the same month more than twice, and correct answers did not fall on the same weekday more than twice. FC made one mistake for the past dates (90% accuracy) and one mistake (90% accuracy) for the future dates. We also asked him 10 reversed questions for past dates (from year 1991 to year 2009) of the type “what are the months having Monday the fifth in 2007?” There were a total of 20 correct answers possible; FC made one mistake and three omissions (80% accuracy).

### COMPUTATION ABILITY

#### Related behaviors

FC can mentally add, subtract, and multiply numbers up to four digits quite rapidly, clearly above his verbal level. His parents reported that FC mastered multiplication tables at the age of seven and knew how to divide numbers at the age of eight. FC then asked for a calculator that he brought with him everywhere. At eight years old, he also showed a great interest in time; he possessed a watch, and knew how to read time.

#### Self report

FC spontaneously explained his method of performing mental calculation. “I calculate with hours, minutes, and seconds, like this it’s easier.” He elaborated: “I did like this (for the addition 1728 + 2932 = 4660): 48 min and 52 s plus 28 min and 28 s equal 1 h 17 min and 40 s.”

#### Test results

Forty numbers were randomly chosen between 1200 and 14,000. The time correspondence of these numbers was then calculated (e.g., 3520 = 58 min and 42 s). For incongruent trials, the time correspondence was modified by ±10 min. (e.g., 3520 = 52 min and 10 s). There were 80 trials: in half (40 trials), numbers were presented with the correct time correspondence whereas in the other half, numbers were presented with an erroneous time correspondence. For each trial, one number and one time (in 24-point Arial font) were presented in the center of a computer screen until FC gave his response by pressing with his right hand the corresponding button “c” for the correct association and “n” for the incorrect association. The experiment was run with PsychoPy software (Peirce, 2007).

FC correctly identified congruent and incongruent associations in 100% of the trials (see **Table 2**). His mean RT for congruent trials (after removing 3SD outliers, which represented 2.56% of the trials) was 6.244 s (SD = 2.14). His mean RT for incongruent trials (after removing 3 SD outliers, which represented 4.87 % of the trials) was 6.725 s (SD = 2.01). There was no difference between congruent and incongruent conditions on RT (*p* > 0.3).

### SYNESTHESIA-LIKE MANIFESTATIONS

#### Related behaviors

FC frequently referred to months of the year to verbalize his emotions or sensations. His parents reported FC saying, for instance, “it hurts like a month of March and half” or that he is happy “like the month of June.” FC can describe his own feeling or emotion with these associations, and asks his parents for an interpretation of certain situations using this month-emotion correspondence (e.g., “like which month are you happy?”). Some numbers can also provoke in FC pleasant sensations.

#### Self report

**FIGURE 2:**
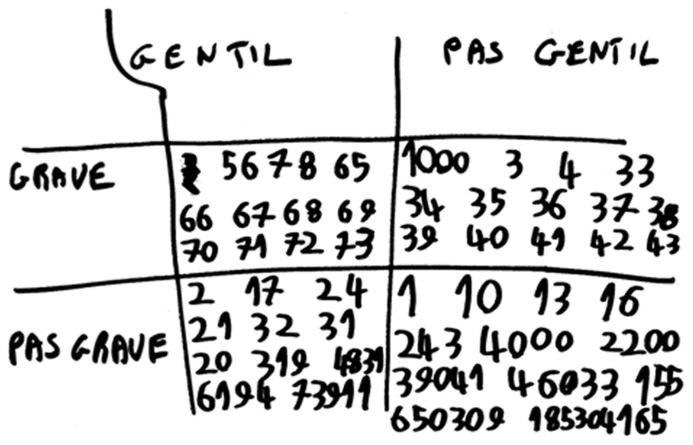
**FC’s representation of numbers according two specific categories: kindness (nice, or “gentil”/ not nice, or “pas gentil”) and mattering (it matters, or “grave”/it does not matter, or “pas grave”)**.

When questioned about the month/emotion correspondence, FC explained that “January is very very bad, February is very bad, March is bad, April is good, May is very good, and June is very very good.” When asked about the number/emotion correspondence, FC reported that some numbers can be “nice or not nice” or that they can “matter or not matter.” In addition, the evocation (or the vision) of certain numbers cause him a physical sensation, as sometimes he reacted to certain numbers as though to a tickle, according to his parents and to our own observations. FC spontaneously provided a graphic representation of how a subset of numbers can be classified as a function of their “kindness” (nice / not nice) and their “mattering” (matters/does not matter; see **Figure 2**). Thus, four categories of emotional valence emerge from FC’s classification. In order to establish a more systematic representation of FC’s categorization of numbers, we questioned him on which emotional valence (i.e., category) each number is associated with. At the beginning, numbers were asked individually and in order. After number 10, only numbers close to the categories’ boundaries were asked. A representation of the architecture of FC’s categorization is given in **Table 3**. FC’s synesthesia-like categories are structured by certain rules. Number of digits in each successive category is multiplied by two (between number 3–4 there are 2 digits, between number 5–8 there are 4 digits, between number 9–16 there are 8 digits, from number17–32 there are 16 digits, and so on…). Categories are presented systematically: *not nice/not matter, nice/not matter, not nice/matter*, *nice/matter*. When FC arrived at the number 8,388,608, he declared he wanted to stop.

**Table 3:** FC’s categorization of numbers.

	Category			
	Not nice, does not matter	Nice, does not matter	Not nice, matters	Nice, matters
1	✓			
2		✓		
3 to 4			✓	
5 to 8				✓
9 to 16	✓			
17 to 32		✓		
33 to 64			✓	
65 to 128				✓
129 to 256	✓			
257 to 512		✓		
513 to 1024			✓	
1025 to 2048				✓
2 049 to 4 096	✓			
4 097 to 8 192		✓		
8 193 to 16 384			✓	
16 385 to 32 768				✓
32 669 to 65 636	✓			
65 537 to 131 072		✓		
131 073 to 262 144			✓	
262 145 to 524 288				✓
524 289 to 1 048 576	✓			
1 048 577 to 2 097 152		✓		
2 097 153 to 4 194 304			✓	
4 194 305 to 8 388 608				✓

#### Test results

Thirty-two numbers of one, two, or three digits were selected at the boundaries of FC’s categories. Numbers were pseudo-randomly selected in the material list in order to avoid the presentation of the same number or same category twice in a row. Each number was presented three times in association with the correct category and three times with the category of the closest number outside the category, for a total of 96 congruent and 96 incongruent trials. For example, in the congruent condition *15* was presented with the correct category *not nice and not matter *and *17* was presented with the correct category *nice and not matter*; whereas in the incongruent condition *15* was presented with the category *nice and not matter* and *17* was presented with the category *not nice and not matter*. The experiment was run with PsychoPy software (Peirce, 2007). In each trial, a number was visually presented with the written name of the congruent or an incongruent category. Numbers and categories were visually presented in the center of the screen and remained presented until a response was given. Number and categories were written in black in 20-point Arial font on a white background, with a 2000 ms blank screen ISI. FC was asked to press a green button for the correct or congruent category and a red button for incongruent categories with his right hand.

FC was 97.9% accurate according to his own classification (see **Table 2**). His four errors were in the incongruent condition (95.8% of correct identification). RTs were also analyzed. Errors and RTs over 3 SD were excluded from analyses (13.5% of trials). A T-test was performed, finding with marginal significance longer RTs for congruent (*M* = 2.331 s, SD = 0.752) compared to incongruent (*M* = 2.041 s, SD = 0.471) trials, *t*(31) = -2.03, *p* = 0.051. However, as noted above, RTs might not straightforwardly reflect FC’s information processing because of his tics and his difficulty in understanding rapid instructions.

## DISCUSSION

This paper provides a naturalistic, empirical, and autobiographical report of a multi-talented autistic savant who is willing and able to comment on his skills. We will now discuss to what extent his savant abilities are related to what is called synesthesia in non-autistic people, how this is beneficial to his performance, and how it supports the role of VM in the acquisition of savant abilities in autism.

### IS FC ACTUALLY A SYNESTHETE?

FC’s abilities can be linked to synesthesia. Indeed, FC’s number/valence attribution bears some resemblance with OLP synesthesia (Simner and Holenstein, 2007). In OLP synesthesia, the inducer is a grapheme, generally a number or a letter, and the concurrent is a specific personality. In some cases, objects can also be associated with a specific personality (Smilek et al., 2007). Persons reporting OLP synesthesia have provided detailed descriptions of the personality associated with graphemes. For example, TE, a 17-year-old woman, reported “Three is such a jerk! He only thinks of himself. He does not care about any other numbers or anything” (Smilek et al., 2007, p. 981). Despite obvious differences in verbal complexity that may be related to differences in verbal IQ, TE’s description is similar to FC’s number/valence associations.

Moreover, FC’s accounts provide evidence that the correspondence between notes and days or months emerged after encountering a specific material during childhood. Synesthesia, as with absolute pitch, is acknowledged to be influenced by childhood experience (Chin, 2003; Simner et al., 2005). This is well-established in the case of the influence of early manipulation of colored alphabets on further grapheme-color synesthesia (Witthoft and Winawer, 2006, 2013). In the case of OLP synesthesia, learning and previous childhood experience might also have shaped the mapping between grapheme and personality types (Simner and Holenstein, 2007; Smilek et al., 2007). Witthoft and Winawer (2013, p. 6) recently argued that synesthesia involves not only perception but also learning and memory: “associative learning and the perceptual experiences of synaesthetes are not only compatible, but also lie on a continuum with ordinary experience.”

Because of FC’s tics and difficulty in understanding rapid instructions, we were unable to provide evidence for the automaticity of his associations, this being one specific characteristic of synesthesia (Simner and Holenstein, 2007). However, we were able to provide evidence that FC’s associations are stable. FC’s associations could still reflect cross-modal correspondences (Spence, 2011), which share certain similarities with synesthesia and pertain to the same continuum (Martino and Marks, 2001). One main point of dissociation is that, unlike synesthetic associations, cross-modal correspondences are not believed to be idiosyncratic. FC’s associations are clearly idiosyncratic. Moreover, automaticity might not be specific to synesthesia, as it has also been observed in cross-modal correspondences (Deroy and Spence, 2013) as well as in induction of verbal notes labels when hearing notes in some absolute pitch possessors. It should be also emphasized that characteristics such as automaticity have been defined in the context of synesthesia occurring in non-autistic people, and therefore that some kind of modification of their nature might be expected when synesthesia occurs in an autistic context. Indeed, the extreme regularity of FC’s associations is not usually observed in synesthesia. This regularity might be the expression of FC’s autism, or just the case of an extreme synesthetic manifestation.

### HOW PERCEPTION HELPS INTELLIGENCE

FC’s way of computing mental additions or multiplications may not appear to be the most economical way, according to typical standards. However, FC declared that this way was easier for him, and it results in accurate operations. This suggests that the correspondence between time units in base 60 up to the level of the second, as well as reciprocal transcoding between base 10 and base 60, is for FC effortless, fast, and stable. A similar gain through fast, cross modal correspondences is found in synesthesia. The ability to map numbers onto a complex combinatorial lexicon of colors and emotions, although out of reach for most people, may result in exceptional memory performances. Rothen et al. (2012) report some cases of exceptional memory in which elements are remembered using variants of the “loci” method. This is the case for Daniel Tammet, an Asperger synesthete with unique learning abilities. Tammet reported that numbers up to an integer of 10,000 have unique shapes, colors, textures, and feels. A list of numbers creates the experience of a complex landscape (Tammet, 2006). The access to such a multimodal experience appears to differ from types of memory facilitation in use in typical individuals. For instance, the chunking of verbal material in memory tasks helps typical individuals, but is not beneficial to Tammet (Bor et al., 2007). Rothen et al. (2012) assumes that Tammet possesses his own internal organization, which helps him to remember long series of digits without interference, but he cannot benefit from a strategy imposed by other minds.

That synesthesia reflects a functional combination of perception, learning, and memory is informative on its role in the acquisition of savant abilities and synesthesia in autism. This perceptual mapping of elements might reflect both an autistic way to create sense from the environment, and a way to feel positive emotions in doing so. Another related possibility would be that FC’s synesthesia allows the coding and further manipulation of pattern-like information naturally presented in a non-discrete form, through its mapping onto mathematical or linguistic structures composed of discrete elements. In this case, synesthesia is used as an investigational tool, top-down or bottom-up or non-hierarchical as necessary, enhancing how an autistic person can manipulate, categorize, and make sense of the world. This is consistent with the enhanced role of perception in intelligence, which represents one of the main messages of neuroimaging studies of autistics (Soulières et al., 2009), and extends far beyond a bottom-up, passive heuristic. It can also be manifested in situations where perceptual structures are used as a creative and investigative tool, for example to crack the codes of emotions and less obviously structured materials.

### VERIDICAL MAPPING AND SAVANT ABILITIES

This report of savant abilities in an autistic adult, FC, benefits from his capacity to document some psychological processes associated to his performances and interests. FC’s performance, his history, and his own intriguing accounts are consistent with the plausible implication of VM, across large-scale isomorphic structures and applied to various materials (numbers, emotions, pitches, musical notation), in the genesis of savant abilities. VM appears to be spontaneous, non-strategic, and bidirectional (or even non-directional), for example between structures that are already represented in the person’s mind, or as a way to collect, organize, understand, and to manipulate new material. Thus this mechanism may contribute to autistic learning, creativity, and everyday information processing.

The notion of veridicality implies that multiple and sufficiently similar occurrences of a structure, across levels and scales, allows the non-hierarchical and non-strategic emergence of a mapping between homologous elements. This is illustrated by FC’s reports on his acquisition of absolute pitch and computation ability. He first associated the seven notes of the musical scale with the seven weekdays, and later on, the 12 semi-tones of the chromatic musical scale with the 12 months of the year. While differing in their substrates, both types of structure share the same number of elements and an ordering constraint, and thereby are highly veridical one to the other. Regarding his mental computation ability, FC computes arithmetical operations initially expressed in base 10 by transposing their constituents into base 60, performing computations in this system, then accurately transposing the result back into base 10. He therefore makes use of isomorphisms between base 60, which structures time measurement, and base 10, which structures arithmetic. This mapping is veridical, in the sense that the same operations can be performed in the two bases, allowing an accurate transformation of an operation expressed in one base to another base. For these two exceptional abilities, the structural similarity is in the object (thereby, is veridical) and not in the eye of the beholder, even if the mapping may appear as an idiosyncratic selection of one structural similarity among multiple others possible.

Critically, FC seems to possess the three main types of abilities – savant syndrome, absolute pitch, and synesthesia – that all, according to our model, plausibly represent an expression of similar or equivalent neurocognitive mechanisms. The micro-structural alterations (Kéïta et al., 2011), enhanced or atypical connectivity (Uddin et al., 2013) involving perceptual areas, and cortical rededication (Samson et al., 2012) characterizing autism could plausibly support a VM mechanism, with various consequences – such as absolute pitch and musical talent, synesthesia, mental computation, calendar calculation – depending on individual variability, availability of materials, and opportunities.

## Conflict of Interest Statement

The authors declare that the research was conducted in the absence of any commercial or financial relationships that could be construed as a potential conflict of interest.

## Footnotes

1 In order to reduce unhelpful biases, and in keeping with the current consensus on language in autism research (see Pellicano and Stears, 2011), we prefer the respectful, accurate term “autistic” rather than “person with autism” (see also Sinclair, 1999).
